# Manzamine A: A promising marine-derived cancer therapeutic for multi-targeted interactions with E2F8, SIX1, AR, GSK-3β, and V-ATPase - A systematic review

**DOI:** 10.1016/j.ejphar.2025.177295

**Published:** 2025-01-23

**Authors:** Mohammad Amir Mishan, Yeun-Mun Choo, Jeffery Winkler, Mark T. Hamann, Dev Karan

**Affiliations:** a Department of Urology, Brown Cancer Center, 505 S Hancock Street, Louisville, KY, USA; b Department of Chemistry, Faculty of Science, University of Malaya, 50603, Kuala Lumpur, Malaysia; c Department of Chemistry, The University of Pennsylvania, Philadelphia, PA, USA; d Department of Drug Discovery and Biomedical Sciences and Public Health, Colleges of Pharmacy and Medicine, Hollings Cancer Center, Medical University of South Carolina, Charleston, SC, USA

**Keywords:** Manzamine A, E2F8, SIX1, AR, GSK-3β, V-ATPases, Cell cycle regulation, Cancer therapy

## Abstract

Manzamine A, a natural compound derived from various sponge genera, features a β-carboline structure and exhibits a range of biological activities, including anti-inflammatory and antimalarial effects. Its potential as an anticancer agent has been explored in several tumor models, both in vitro and in vivo, showing effects through mechanisms such as cytotoxicity, regulation of the cell cycle, inhibition of cell migration, epithelial-to-mesenchymal transition (EMT), autophagy, and apoptosis through multi-target interactions of E2F transcriptional factors, ribosomal S6 kinases, androgen receptor (AR), SIX1, GSK-3β, v-ATPase, and p53/p21/p27 cascades. This systematic review evaluates existing literature on the potential application of this marine alkaloid as a novel cancer therapy, highlighting its promising ability to inhibit cancer cell growth while causing minimal side effects.

## 1. Introduction

Cancer, being one of the leading causes of death worldwide, continues to be formidable across the globe (Siegel et al., 2024). Over the decades, there has been a significant advancement in developing new treatment therapies. Unfortunately, some cancer cells manage to slowly acquire resistance to the drug, developing complementary survival signaling pathways or genetic and epigenetic alterations to evade the immune system (Khan et al., 2024). Once the cancer cells metastasize (spread to the other organs from its origin), it becomes even more challenging to treat due to the lack of additional new therapies as molecular targets of the evolving new variant cells differ significantly (Qian et al., 2017). The plasticity of the cellular and molecular events in the initiation, development, and progression of cancer cells represent the complexity of the interconnected cascades. However, several of the basic mechanisms involved in cancer progression include epithelial-to-mesenchymal transition (EMT), cell cycle progression, autophagy, and resistance to apoptosis (Debnath et al., 2023; Matthews et al., 2022; Neophytou et al., 2021; Singh and Siddique, 2023). Overcoming each one of these processes could be considered a new therapeutic strategy for cancer.

EMT is a fundamental process in cancer cell migration and metastasis. During EMT, epithelial cells transform and adopt the characteristics of mesenchymal stem cells for migrating and establishing colonies in distant organs (Bakir et al., 2020; Roche, 2018). EMT has been implicated in various aspects of cancer development, including drug resistance or chemoresistance in several cancer types. Many EMT-associated transcription factors (TFs), such as Zeb1, Twist, Snail, and Slug, along with EMT-inducing signaling pathways like transforming growth factor-beta (TGF-β) and Wnt, have been shown to play an essential role in promoting drug resistance in cancer cells (Ashrafizadeh et al., 2024; Ebrahimi et al., 2024; Guo et al., 2024; Xue et al., 2024). Moreover, autophagy, a conserved cellular mechanism that degrades damaged biomolecules, is closely connected with EMT primarily through activating the energy response pathways or modulating the degradation of EMT-related adhesion and cytoskeletal molecules such as E-cadherin (Hwang et al., 2022; Strippoli et al., 2024; Zada et al., 2019). Therefore, numerous studies have concentrated on inhibiting this process as a strategy to combat cancer, and drugs targeting EMT represent a valuable therapeutic avenue.

On the other hand, autophagy plays a dual role in cancer as a tumor suppressor by preventing the accumulation of damaged proteins and organelles or functioning in cell survival by recycling organelles to facilitate energy production, thereby promoting tumor growth (Qu et al., 2003). Autophagy is also implicated in chemotherapy resistance by removing the drug effect (Sui et al., 2013). In addition, tumor cells develop resistance to apoptosis through various molecular mechanisms, such as dysregulation of the mitochondrial pathway, inactivation of caspases, and a lack of death signals through TNF superfamily death receptors (Fulda, 2010; Olsson and Zhivotovsky, 2011). The use of conventional therapies, characterized by various side effects, drug resistance, and limited accessibility, poses significant hurdles in cancer treatment (Nikolaou et al., 2018). Thus, additional new treatment approaches are essential to overcome these barriers and enhance the overall effectiveness of cancer therapies.

Considering these challenges, natural products with anticancer properties such as terpenoids and volatile oils (paclitaxel), polyketides (etoposide), alkaloids (vincristine), quinones (tanshinone), etc., have proven to be highly successful due in part to their unique structural features and complexity, which help aid in target selectivity and potential off-target interactions. In this context, marine organisms have emerged as a novel source of naturally occurring alkaloids that show promise in combating cancer metastasis (Tohme et al., 2011). Manzamine alkaloids, which are β-carboline compounds featuring a polycyclic ring, are extracted from sponges belonging to the genera *Haliclona, Pellina, Xestospongia, Ircinia*, and *Pachipellina* (Edrada et al., 1996; Ichiba et al., 1994; Watanabe et al., 1998). Manzamine A (MA), initially isolated from *Haliclona sp*. in the Okinawa Sea, was reported to exert an anti-proliferative effect on mouse leukemia cells (Sakai et al., 1986). Additionally, it has demonstrated diverse bioactivities, including immunostimulation (Ang et al., 2001), anti-inflammatory effects (Mayer et al., 2005; Yousaf et al., 2004), antiviral (Palem et al., 2017), antimalarial activity (Peng et al., 2010), neuritogenic potential (Zhang et al., 2008), and suppression of hyperlipidemia and atherosclerosis (Eguchi et al., 2013; Shilabin et al., 2008). Therefore, this systematic review compiles evidence from the literature concerning the mechanistic effects of MA on human cancer cells, with a focus on cell cytotoxicity, inhibition of the cell migration/EMT process, apoptosis, and autophagy through multi-target interactions, including E2F8, SIX1, AR, GSK-β, and V-ATPases.

## 2. Methods

### 2.1. Systematic literature search

**Fig. 1.**
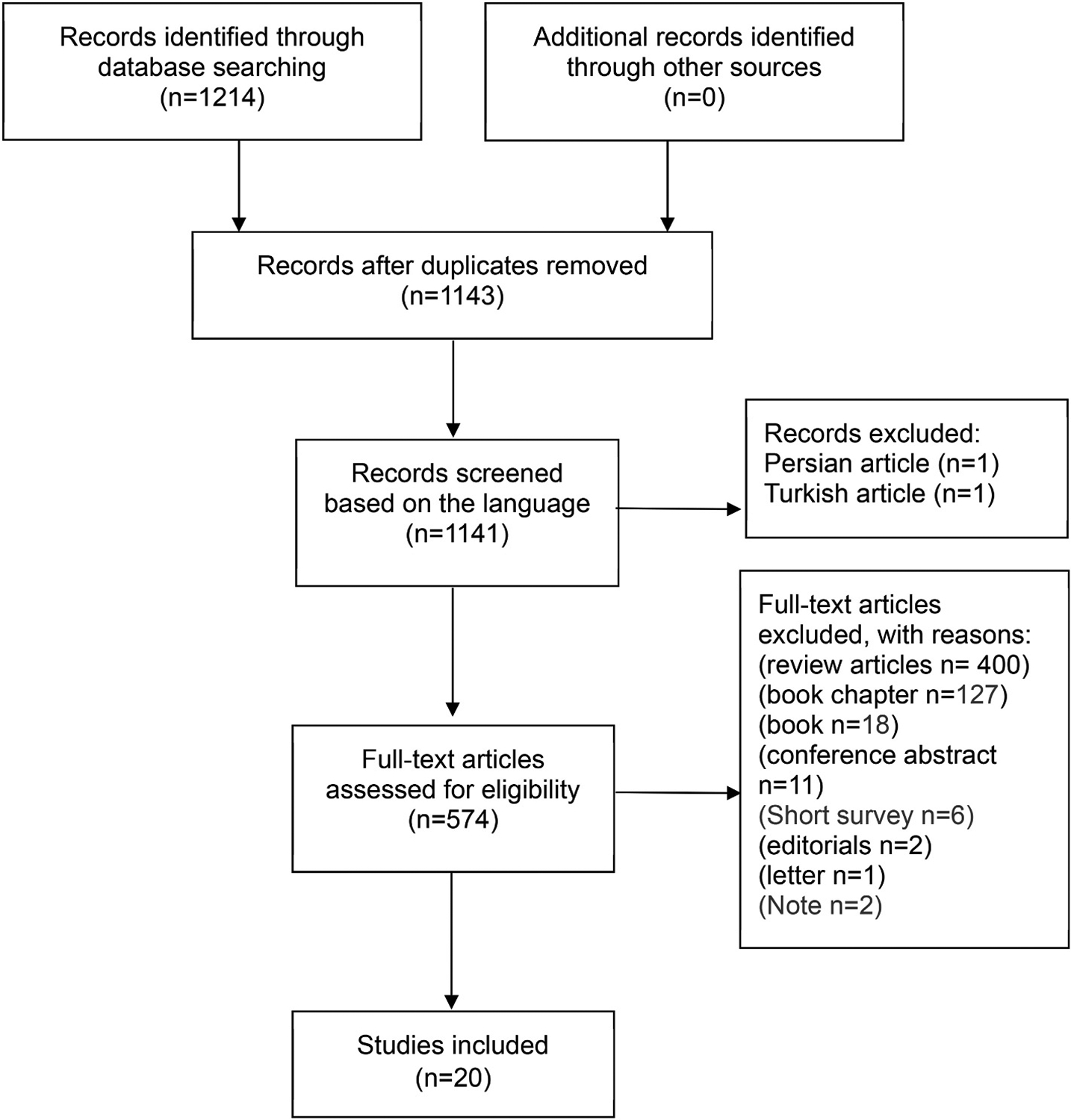
Flow diagram of the study selection process on using MA as a potential therapeutic targeting cancer.

We conducted a comprehensive literature review, examining qualified papers available in PubMed, Scopus, Cochrane Library, and Web of Science (ISI) databases up to February 20, 2024. The search utilized specific keywords: (tumor OR neoplasm OR malignant OR cancerous OR cancer OR neoplasia OR malignancy in combination with Manzamine A or marine sponge), based on which we identified 574 publications. Subsequently, the identified articles underwent screening, and the collected literature was reported following the guidelines outlined in the PRISMA statement for systematic reviews and meta-analyses (Liberati et al., 2009) and focused on 20 studies (Fig. 1).

### 2.2. Eligibility criteria

After the initial search, titles were checked for duplicates. Studies underwent evaluation for eligibility based on the relevance of titles and abstracts to the topic, and the selected items were compared to remove the redundant cases. This study encompassed all English-published papers on human cancer cells, regardless of the publication date, including those involving in vitro, in vivo, and computational approaches. We retrieved the full text to extract essential data. Exclusion criteria comprised duplicative studies, non-English articles, review articles, conference papers, letters, book chapters, meeting abstracts, short surveys, unnecessary articles, and editorials.

## 3. Results

### 3.1. Mechanistic details on the anticancer potential of Manzamine A

The screening criteria identified 20 studies relevant to the anticancer activity of manzamine A (MA) and related marine compounds (Table 1). The cytotoxic effects of MA and other marine compounds (e.g., axinastatin 5, majusculamide C, jaspamide, bengazoles A, B, and E, neoechinulin A, and 8-hydroxymanzamine A) on several cancer cells were reported in two studies (Pettit et al., 2008a; Samoylenko et al., 2009a). Among all the studies, two articles focused on the inhibitory effects of MA on the EMT process in colorectal and breast cancer by downregulating mesenchymal markers and upregulating epithelial markers (Lin et al., 2018; Wang et al., 2023). In two other studies, MA showed suppression effects on autophagy in pancreatic and breast cancer (Kallifatidis et al., 2013; Wang et al., 2023). In pancreatic cancer, 10 μM MA affected vacuolar ATPase activity in the cells and increased the LC3-II autophagosome marker as well as p62/SQSTM1 (Kallifatidis et al., 2013). Similarly, in breast cancer cells, MA showed a suppressive effect on autophagy by blocking autophagosome-lysosome fusion and reducing autophagosome degradation (Wang et al., 2023).

The cell cycle arrest as a potential inhibitory effect of MA and its analogs has been demonstrated in several cancers (Karan et al., 2020; Ko et al., 2019; Lin et al., 2018, 2022; Tu et al., 2004). In colorectal cancer, MA treatment downregulated the expression of E2F transcription factor and induced cell cycle arrest at the G0/G1 phase by decreasing the expression of CDK2/4 and cyclin D1 through the p53/p21/p27 pathways (Lin et al., 2018). Likewise, MA blocked cell cycle progression at the G1/S phase and induced p53/p21 expression in cervical cancer cells by 4 μM concentration. SIX1, an oncoprotein and transcription factor with high expression levels in cancer, is upstream of p53 and downstream of E7, and CK2α was downregulated in cervical cancer cells following MA treatment (Karan et al., 2020).

In one of the earlier studies, MA decreased the gene expression of p53 in breast cancer cells by an effective dose of 50 μM without any effects on p16 and p21 levels (Arif et al., 2004). It was somewhat unusual that MA was effective at a high concentration of 50 μM. Although there can be lab-to-lab variations, discrepancy at such a high concentration may be due to the lower purity of MA or difficulties related to the solubility of the compound. Recently, we demonstrated that MA (3–6 μM) suppressed the androgen receptor (AR) biosynthesis and AR-regulated genes by blocking the E2F8 transcription factor, and E2F8 was identified as a potential therapeutic target of the MA drug. In addition to apoptosis induction and cell cycle gene regulation in prostate cancer cells, MA remarkably inhibited prostate tumor growth in mice (Karan et al., 2024). For in vitro studies, we successfully resuspended the MA drug in DMSO at low concentrations. However, preparing a high dose of the MA drug in DMSO for in vivo studies and achieving solubility was a challenge. After testing several solvents and their combinations, we discovered that adding 10–30 μL of HCl to the MA suspension in DMSO resulted in a complete solution. We then adjusted the remaining volume of the MA drug suspension to 1 mL with DMSO to achieve the desired dose. This drug solution was aliquoted into 100 μl each and stored at −20 °C until needed for treatment by oral gavage. On the day of treatment, a 100 μl aliquot was resuspended in 900 μL of filter-sterilized 0.9% NaCl solution, and 100 μL of MA drug was administered at a dose of 30 mg/kg of body weight. In our preclinical study, tumors were established in nude mice using subcutaneous injection of enzalutamide-resistant 22Rv1 prostate cancer cell lines. Following the development of palpable tumors, the mice were given an oral dose of MA at 30 mg/kg/BW twice or thrice per week. MA significantly inhibited the growth of chemo-resistant 22Rv1 tumor xenografts while maintaining the average body weight of the mice, with no signs of cytotoxicity observed in the histopathological examination of major organs or the blood chemistry of liver panel enzymes. These results suggest that MA has the potential as a drug candidate to overcome therapy resistance cancer.

Five studies have focused on and provided evidence of the ability of MA to induce apoptosis in cancer cells (Guzmán et al., 2011; Lin et al., 2018, 2022; Ougolkov et al., 2005; Yadav et al., 2014). MA exhibited apoptosis induction in pancreatic cancer cells by inhibiting glycogen synthase kinase-3 beta (GSK-3β) and NF-κB signaling (Ougolkov et al., 2005). Also, MA inhibited single cell formation and sensitized pancreatic cancer cells to tumor necrosis factor (TNF)-related apoptosis-inducing ligand (TRAIL)induced cell apoptosis (Guzmán et al., 2011). In colorectal cancer cells, MA induced apoptosis by upregulating AP-1 and ATF transcriptional factors, causing mitochondrial membrane deformation (Lin et al., 2018). In a study on glioblastoma, MA unveiled inhibitory effects on GSK-3β and its downstream pathways, splicing factors, PTBP1, SF2/ASF, and hnRNPA1, as well as anti-apoptotic regulators. Interestingly, an upregulation of Anxa7 gene expression was also observed following MA treatment (Yadav et al., 2014).

**Fig. 2.**
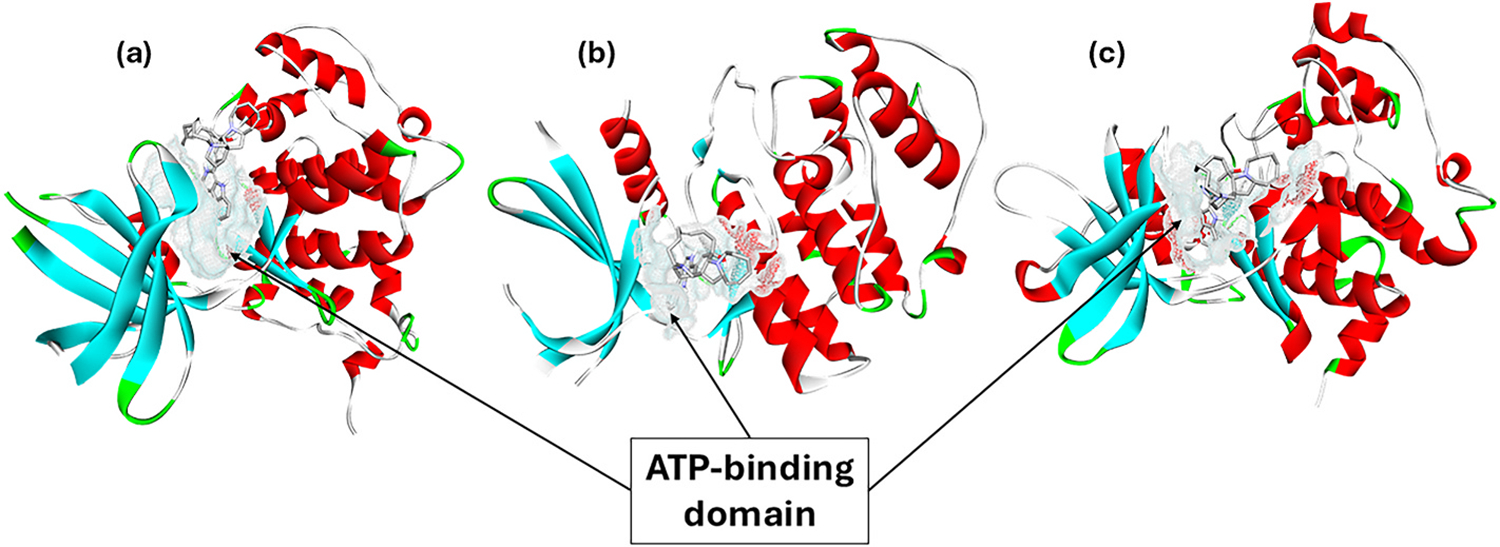
MA binds at the ATP-binding domain of (a) GSK-3β, (b) CDK-5, and (c) CK2α. The figure was created by YM Choo using the BIOVIA, Dassault Systèmes, Discovery Studio Visualizer software (version 21.1.0.20298), which was employed to perform the calculations and produce the graphical results.

The inhibitory effect on kinases was identified as another impact of MA on cancer cells. MA inhibited GSK-3β and cyclin-dependent kinase 5 (CDK-5) with IC_50_ values of 10 and 1.5 μM, respectively (Hamann et al., 2007) (Fig. 2). GSK-3β and CDK-5 are pivotal for cell cycle progression, and blocking these kinases induces cell cycle arrest and apoptosis. In a separate investigation, MA underwent screening against 30 protein kinases, revealing a 68% reduction in the activity of rat p90 ribosomal S6 kinase 1 (RSK1) at a concentration of 1 μM. Table 2 summarizes the inhibition activity of MA on protein kinases, where an inhibition of 10% or more is observed (Mayer et al., 2021). The study also revealed the substantial effectiveness of MA in suppressing the 90 kDa ribosomal S6 kinase (RSK)1/2, particularly RSK1, in human cervical carcinoma cells (with relative IC_50_ values of 15.01 μM for RSK1 and 108.4 μM for RSK2). The computational analysis revealed that MA preferably binds to the N-terminal kinase domain (NTKD) of RSK1 over the C-terminal domain (CTKD). The predicted binding energies for the NTKD and CTKD complexes with MA were −62.132 and −55.497 kcal/mol, respectively (Mayer et al., 2021).

**Fig. 3.**
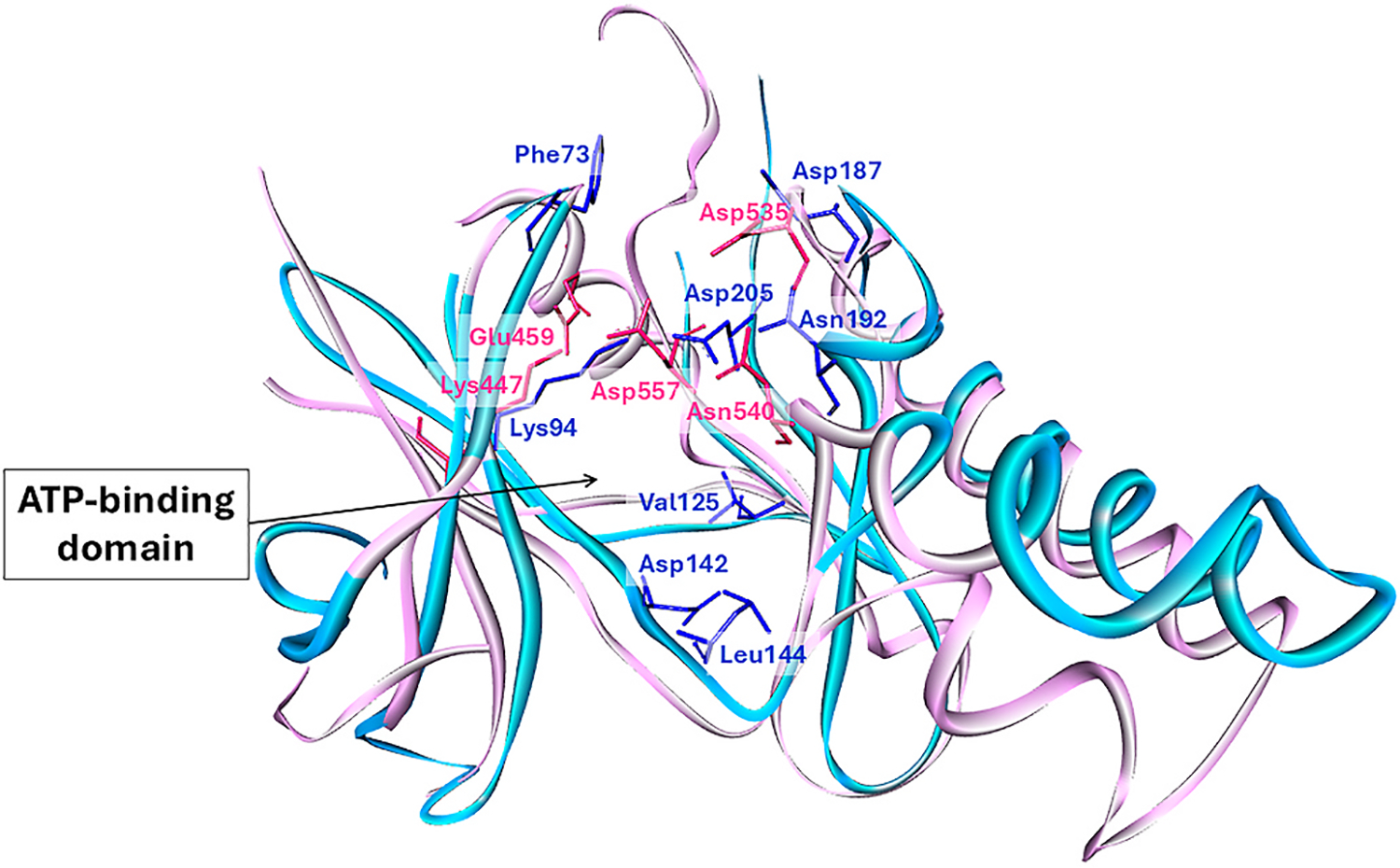
Interacting amino acid residues at the ATP-binding domain overlayed NTKD RSK1 (blue) and CTKD RSK1 (pink). The figure was created by YM Choo using the BIOVIA, Dassault Systèmes, Discovery Studio Visualizer software (version 21.1.0.20298), which was employed to perform the calculations and produce the graphical results.

The ribosomal S6 kinase (RSK) family consists of cytosolic serine-threonine kinases that play crucial roles downstream of the Ras/ERK1/2 pathway and regulate cell growth, survival, and proliferation by phosphorylating substrates (Romeo et al., 2012a). These kinases comprise two functional domains: the C-terminal kinase domain (CTKD), which ERK1/2 phosphorylates, and the N-terminal kinase domain (NTKD), responsible for substrate phosphorylation (Romeo et al., 2012b). Significantly, sustained RSK activation is associated with cancer (Cho et al., 2007; Redman et al., 2013). Computational analysis predicted that MA binds preferentially to the ATP binding pocket of NTKD RSK1. The study noted that MA establishes more robust interactions with amino acid residues and binds deeper into the binding domain of NTKD RSK1 compared to CTKD (Fig. 3). In vitro studies using human cervical carcinoma cells revealed that MA effectively inhibited the RSK1 and RSK2 with a high potency toward RSK1 (Mayer et al., 2021).

Anti-cell proliferation, a critical effect of MA, was shown on leiomyoma cells by targeting sterol o-acyltransferases (SOATs), blocking cholesterol esterification and the accumulation of free cholesterol, which induced unfolded protein response (UPR) sensors, PRK-like endoplasmic reticulum kinase (PERK), inositol-requiring enzyme (IRE1), and activating transcription factor (ATF6), leading to endoplasmic reticulum (ER) stress-induced cell death (Lin et al., 2023).

Five recent studies have demonstrated the synthesis and characterization of MA-inspired structures (Ko et al., 2019; Lin et al., 2022; Shen et al., 2005, 2011; Tu et al., 2004). One study compared the cytotoxicity and cell cycle arrest ability of MA with its analog, Mana-Hox (Tu et al., 2004). Mana-Hox blocked cell division at the M phase without affecting the earlier steps in the cell cycle transition. A similar study synthesized 1-substituted 1,2,3,4-tetrahydro- and 3,4-dihydro-β-carboline derivatives, demonstrating its cytotoxic effects on various cancer cells through chromosome missegregation (Shen et al., 2005). Another MA derivative, 1-(9′-propyl-3′-carbazole)-1, 2, 3, 4-tetrahydro-β-carboline (PCTC), induced apoptosis in glioma cells by increasing phosphorylated JNK (p-JNK) and phosphorylated p38 (p-p38) associated with intracellular reactive oxygen species (iROS) generation (Lin et al., 2022). This effect was accompanied by the upregulation of caspase 3/7, elevated enzymatic activity of PARP, and downregulation of the anti-apoptotic protein Bcl-2. Moreover, combining PCTC and temozolomide (TMZ) showed a synergistic effect via blocking cell cycle progression in glioma cells (Lin et al., 2022). Similarly, two Manzamine-derived synthetic compounds, 1-substituted carbazolyl-1, 2, 3, 4-tetrahydro-β-carboline and carbazolyl-3, 4-dihydro-β-carboline, exhibited inhibitory effects on colon, lung, and hepatoma cells (Shen et al., 2011). Additionally, 1-(9′-methyl-3′-carbazole)-3, 4-dihydro-β-carboline (MCDC), another synthetic compound, demonstrated inhibitory effects on macrophage migration inhibitory factor (MIF), AKT phosphorylation, and the expression of S phase-related proteins in breast cancer cells (Ko et al., 2019).

**Fig. 4.**
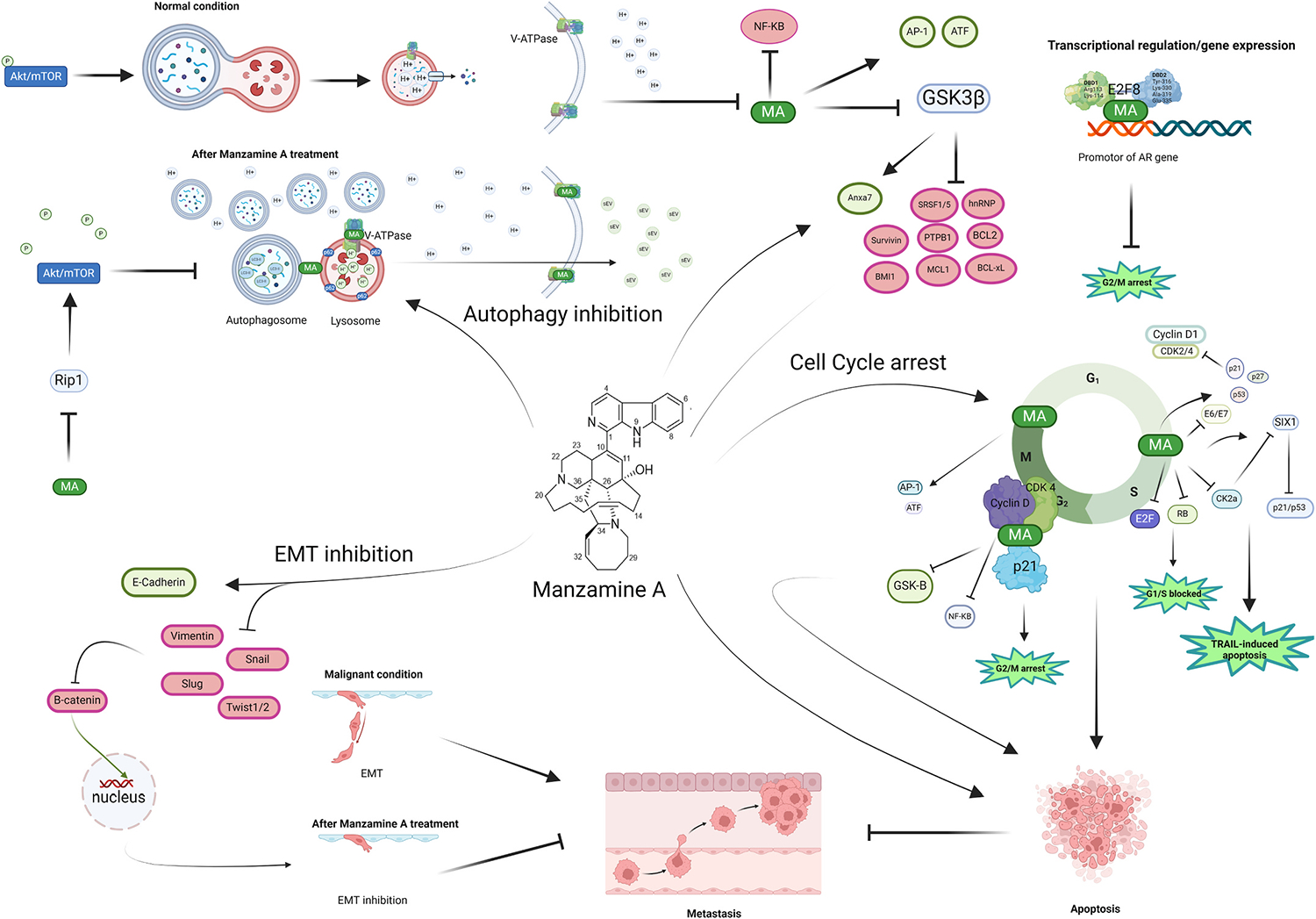
Mechanisms of MA with multi-targeted interactions and anticancer potential in targeting cancer. MA showed inhibitory effects on the cell cycle regulators, suppression of the cell transition from epithelial to mesenchymal (EMT) in cancer cells, autophagy inhibition mediated via blocking autophagosome-lysosome fusion, and transcription inhibition of E2F8. This figure was generated using BioRender illustration software.

Computational analysis revealed nine small molecules (Cyclosporin A, MA, Cardidigin, Staurosporine, Benzo [a]Pyrene, Sitosterol, Nocardiopsis Sp, Troglitazone, and Riccardin D) as the top-ranked candidate drugs for treating colorectal cancer (Horaira et al., 2023). Similarly, in another study, seven drugs (MA, Cardidigin, Staurosporine, Sitosterol, Benzo [a]pyrene, Nocardiopsis sp., and Riccardin D) were reported for colorectal cancer based on affinity in molecular docking. Among these candidate drugs, MA remains a highly effective drug molecule (Islam et al., 2023). The molecular mechanisms of MA on cancer cells are displayed in Fig. 4.

The unique structure of MA, which embodies a β-carboline hetero-cycle attached to a novel pentacyclic diamine core containing both eight- and thirteen-membered rings on a pyrrolo [2,3-*i*]isoquinoline framework, has stimulated considerable interest and activity directed toward its total synthesis of MA analogs. These studies culminated in the first total synthesis of MA analogs in 1998 by Winkler and Axten. Since that time, syntheses of MA analogs have been reported by several groups (Humphrey et al., 2002; Jakubec et al., 2012). The development of MA analogs with improved therapeutic efficacy could benefit drug combinations to manage drug-resistant cancers in patients who have exhausted other treatment strategies. However, none of the reported syntheses are sufficiently efficient to produce the required amounts of MA analogs for a drug development program, highlighting the importance of further work in this area.

## 4. Discussion

Epithelial-mesenchymal transition (EMT) is an initial process and one of the essential steps of cancer cell migration and metastasis (Mittal, 2018; Singh and Siddique, 2023). Therefore, anti-EMT compounds may serve as critical drug targets. In this context, MA was shown to induce E-cadherin while reducing Snail, Slug, and Twist1/2 expression, preventing the nuclear translocation of β-catenin, leading to the restoration of tight and adherent junctions between epithelial cells in colorectal cancer cells (Lin et al., 2018). A similar effect was observed in breast cancer, where MA abolished the migration of cancer cells by influencing the EMT process through the elevation of E-Cadherin and the reduction of mesenchymal markers, Vimentin and Snail (Wang et al., 2023). Notably, MA showed the ability to decrease claudin-1 expression, an integral membrane protein with elevated expression in metastatic colon cancer cells (Lin et al., 2018). Claudin-1 is known to regulate cellular transformation and metastasis by impacting E-cadherin and β-catenin/TCF signaling pathways (Dhawan et al., 2005).

Tumor cells adapt to propagate in the acidic conditions of the tumor microenvironment (TME) (Chung et al., 2011), and vacuolar ATPases (v-ATPases) facilitate such adaptive response by using the energy released during ATP hydrolysis to transport protons from the cytoplasm to cellular compartments like lysosomes, thereby creating an alkaline pH in the cytoplasm (Sennoune et al., 2004; Spugnini et al., 2010). Lack of accumulation of H^+^ ions in tumor cell cytoplasm prevents the activation of lytic enzymes that are typically active at lower pH levels (Fais et al., 2007). V-ATPases are also present in the plasma membrane of cancer cells like pancreatic cancer cells, actively transporting protons from the cytoplasm to the external space (Chung et al., 2011). Blocking v-ATPases using MA led to the buildup of protons in the cytoplasm, ultimately causing an elevation in cytosolic acidity (Kallifatidis et al., 2013). Thus, MA can potentially suppress the tumor-promoting physiological conditions in the TME.

During the autophagy process, the p62 protein, also known as sequestosome 1 (SQSTM1), has a rapid degradation rate in physiological conditions in the lysosomal compartment (Mathew et al., 2009). A potential molecular link between defective autophagy and tumorigenesis involves the accumulation of p62/SQSTM1 protein aggregates and damaged mitochondria, producing reactive oxygen species (ROS) that cause DNA damage (Mathew et al., 2009). Treatment with MA demonstrated an accumulation of LC3-II autophagosome marker and increased p62 levels in pancreatic and breast cancer cells, indicating a disruption in autophagosome turnover (Kallifatidis et al., 2013; Wang et al., 2023). Consequently, MA obstructed autophagy at the level of autophagosome-lysosome fusion and autolysosome degradation, resulting in the accumulation of autophagosomes, acidic lysosomes, and autolysosomes (Kallifatidis et al., 2013). This pattern closely resembles the effects observed with bafilomycin A1 (Kallifatidis et al., 2013). However, MA demonstrated better in vivo tolerance than bafilomycin A1 (Ang et al., 2001; Dröse and Altendorf, 1997). Subsequently, the accumulation of autophagic vacuoles resulting from inhibiting v-ATPases and autophagy is associated with enhanced cell death triggered by lysosomal dysfunction (Kroemer and Jääattelä, 2005; Nakashima et al., 2003). Given the indispensability of autophagy for pancreatic tumor growth, the blockade of this pathway by MA suggests a promising strategy for targeting pancreatic cancer. A similar effect of MA was observed on breast cancer cells by augmenting autophagosome accumulation and simultaneously reducing autophagosome degradation through a decrease in lysosomal acidity (Wang et al., 2023). Besides, MA induced the release of small extracellular vesicles (sEVs) rich in autophagy-related proteins from breast cancer cells, enhancing secretory autophagy (Wang et al., 2023).

A key regulator of autophagy is the mammalian target of the rapamycin (mTOR) pathway (Pattingre et al., 2008), which is activated downstream of PI3K-AKT, a pathway commonly dysregulated in cancer (Semenza, 2010; Takeuchi et al., 2005). TOR kinase is a major autophagy inhibitor, suppressing the autophagy process in response to growth factors and insulin-like signals during nutrient abundance (Thorburn, 2008). In this context, mTOR inhibitors, such as rapamycin, have demonstrated the induction of autophagy in tumor cells (Jung et al., 2009; Takeuchi et al., 2005). MA downregulated the phosphorylation of AKT and mTOR by decreasing the expression level of RIP1, the key upstream regulator of mTOR/AKT, in breast cancer cells (Wang et al., 2023).

Intracellular reactive oxygen species (iROS) play a significant role in mitogen-activated protein kinase (MAPK) signaling activation, which is crucial for cellular function (Faghfuri et al., 2018; Zhao et al., 2015). The MAPK subfamilies, including ERK, JNK, and p38 kinases, mediate apoptosis and autophagy in response to various stimuli through their downstream effectors (Cargnello and Roux, 2011; Sui et al., 2014). PCTC, a derivative of MA, elevated the levels of p-JNK and p-p38, which are associated with iROS generation, ultimately leading to cell apoptosis in glioma cells by augmenting caspase 3/7, PARP, and repressing the level of the anti-apoptotic protein Bcl-2 (Lin et al., 2022). TMZ exerts its effect by inducing G2/M cell cycle arrest and eventual apoptosis (Zhang et al., 2012). On the other hand, PCTC-induced cell cycle arrest was observed at the G1/S transition (Lin et al., 2022). Therefore, the synergistic effect of PCTC in combination with TMZ might be attributed to the simultaneous blockage of different cell cycle phases.

The E2F family and their heterodimerization partner proteins, such as retinoblastoma (Rb), serve as transcriptional regulators of the G1/S transition, with their dysregulation being frequently dominated in cancer. Also, the phosphorylation of E2F pocket proteins by cyclin D and CDK4/6 promotes the transcription of G1/S target genes, including cyclin E. This leads to subsequent phosphorylation of their pocket proteins by cyclin E/CDK2, forming a positive feedback loop that assists cell cycle progression (Bertoli et al., 2013; Trimarchi and Lees, 2002). MA induced E2F downregulation in colorectal cancer cells while inducing cell cycle arrest at the G0/G1 phase by lowering the expression of CDK2/4 and cyclin D1 and impacting p53/p21/p27 pathways (Lin et al., 2018).

Pancreatic cancer cells frequently display activated NF-κB, associated with metastasis and apoptosis resistance (Fujioka et al., 2003). It was found that the upregulation of nuclear GSK-3β is associated with dedifferentiation and NF-κB-mediated survival in pancreatic cancer, and MA significantly inhibited NF-κB and GSK-3β (Ougolkov et al., 2005). The cytotoxicity of MA is linked with the β-carboline-mediated intercalation, and the β-carboline stabilizes associations with GSK-3β-binding (Waters et al., 2010). Dysregulation of GSK-3β is tissue-specific, with several cancers showing elevated expression of its active form (Kitano et al., 2013; Ougolkov et al., 2005). MA inhibited the activation of tyrosine phosphorylation of GSK-3α(Tyr 279)/β(Tyr 216), and affected the translocation of GSK3 kinase, β-catenin, and pERK1/2 to the nucleus in glioblastoma cells (Yadav et al., 2014).

Several splicing factors, like polypyrimidine tract binding protein (PTBP1), SR protein (SRSF1), and heterogeneous nuclear ribonucleo-proteins (hnRNPs), are highly expressed in cancer (LeFave et al., 2011; Manley and Krainer, 2010). SRSF1 regulates the expression of the tumorigenic tyrosine kinase receptor (RON) and is allied to shorter survival in glioblastoma patients (Ghigna et al., 2005). It is proposed that p38 kinase-mediated upregulation of nuclear hnRNPA1 in senescent fibroblast with inhibition of nucleocytoplasmic shuttling (Shimada et al., 2009). Treatment of glioblastoma cells with MA led to the inhibition of GSK3 kinase, resulting in the downregulation of SRSF1/5, PTPB1, and hnRNP with decreased levels of anti-apoptotic genes such as *BCL2, BCL-xL, Survivin, MCL1*, and *BMI1* while increased in *Anxa7*, a tumor suppressor gene (Yadav et al., 2014).

Increased activity and expression of sterol *O*-acyltransferases (SOATs) have been observed in human colon carcinoma and hepatocellular carcinoma (HCC), and the inhibition of SOAT2 is shown to suppress HCC tumor cell growth (Lu et al., 2013; Song et al., 2006). Interestingly, MA exhibited an anti-proliferative effect on uterine leiomyoma cells by targeting SOAT, which impaired cholesterol esterification, leading to ER stress-induced cell death by provoking UPR sensors, PERK, IRE1, and ATF6. Also, MA impeded the expression of nuclear β-catenin, a downstream target of SOAT (Lin et al., 2023).

In human papillomavirus (HPV) driven cancers, E6 and E7 oncogenes disrupt cell-cycle regulation. In this context, E6 promotes p53 degradation, while E7 is associated with the phosphorylation of Rb proteins (Huh et al., 2007; Talis et al., 1998). Both *p53* and *Rb* are vital tumor suppressor genes in maintaining cell-cycle regulation. The interaction between cyclin D1 and Rb protein positively correlates with tumorigenesis (Arif et al., 2004). Following MA treatment, an increase in p53 and p21 protein levels was observed in cervical cancer cells, along with the inhibition of cyclin D1, total Rb, and p-Rb, suggesting that MA may influence the regulation of both E6 and E7 oncogenes, thereby facilitating anticancer activities (Karan et al., 2020).

Similarly, SIX1, a transcription factor containing a homeodomain, is overexpressed in various cancers, leading to downregulating p53 with aggressive clinical behavior and poor outcomes (Bian et al., 2024; Coletta et al., 2004; Reichenberger et al., 2005; Towers et al., 2015). Notably, E7 oncogene stimulates SIX1 expression in cervical intra-epithelial neoplasia and cervical cancer cells and accumulation of cyclin D1 in tumor cells, resulting in tumorigenesis (Liu et al., 2014). In addition to E7 oncogene, SIX1 contains conserved CK2 sites and is regulated by CK2 protein kinase (Ford et al., 2000; Liu et al., 2014). CK2 inhibition resulted in decreased phosphorylation of SIX1, suggesting SIX1 is a potential therapeutic target. Molecular docking studies showed that MA binds favorably to the ATP-binding domain of CK2α (Fig. 3), consistent with findings from the protein kinase screening study, demonstrating a 17% reduction in CK2α activity when treated with 1 μM of MA (Table 2) (Karan et al., 2020; Mayer et al., 2021). MA repressed CK2α protein expression with subsequent inhibition of the SIX1 protein (Karan et al., 2020).

## 5. Conclusion and perspective

Natural products are an extraordinarily significant source of unique classes of lead prototypes for controlling various diseases, including cancer. The marine sponge product MA has demonstrated neuro-protective, anti-inflammatory, anti-microbial, and antiviral properties with remarkable potency against drug-resistant strains of the malaria parasite, *Plasmodium* spp. MA also showed repressive effects on several types of cancer by inhibiting cell cycle regulation, autophagy process, and EMT while inducing an apoptotic cascade. Specifically, MA has been shown to downregulate mesenchymal markers in colorectal and breast cancer, while the effects on the vacuolar-ATPase activity cause the suppression of autophagy in pancreatic cancer. Additionally, MA can inhibit apoptosis and autophagy through multi-target interactions, including E2F8, SIX1, AR, v-ATPases, as well as through kinases such as GSK-3β, CDK-5, and ribosomal S6 kinases (RSK1/2) in breast, prostate, pancreatic, and colorectal cancer. All these events are critical for tumor development, progression, and cancer cell metastasis. The unique multitargeted effect of MA demonstrates significant promise in reducing cancer cell proliferation and opens the door to innovative drug combinations. Moreover, our recent observation shows that the MA is a highly effective inhibitor of E2F8 with subsequent repression of AR. This has proven to be highly effective in prostate cancer models for inhibiting the growth of drug-resistant tumors in mice. Thus, MA shows promise across various therapeutic areas and could be considered a novel therapeutic targeting cancer. The compelling anticancer effectiveness of MA in both in vitro and in vivo studies supports the development of potential clinical trials in the future.

## Figures and Tables

**Table 1:** The main features of the twenty studies included in this systematic review.

Type of Study (References)	Type of Cancer	IC_50_	Identified Potential Anticancer Effect and Mechanism
In vitro/synthetic (Tu et al., 2004)	Multiple cancer types and cell lines	48h: 1.3–5.4 (μM) 72h: 2.4–6.2 (μM)	Cytotoxicity
In vitro (Arif et al., 2004)	Breast cancer	50 μM	P53 downregulation
In vitro (Shen et al., 2005)	Multiple cancer types and cell lines	0.03–0.1 (μg/ml)	Cytotoxicity
In vitro (Pettit et al., 2008b)	Multiple cancer types and cell lines	–	Cytotoxicity
In vitro (Samoylenko et al., 2009a)	Multiple cancer types and cell lines	–	​
In vitro (Samoylenko et al., 2009b)	Multiple cancer types and cell lines	1–4.4 (μg/ml)	Cytotoxicity
In vitro (Guzmán et al., 2011)	Pancreatic cancer	48h: 40 (μM) 72h: 4.2 (μM)	Various cellular assays
In vitro (Shen et al., 2011)	Multiple cancer types and cell lines	–	Inhibition of human DNA topoisomerase II
In vitro (Kallifatidis et al., 2013)	Pancreatic cancer	–	Autophagy inhibition
In vitro (Yadav et al., 2014)	Glioblastoma	–	Apoptosis
In vitro (Lin et al., 2018)	Colorectal cancer	24h: 4.5->10 (μM)	Various cellular assays
In vitro/computational (Ko et al., 2019)	Lung and Breast cancer	48h: 5 (μM)	Cell cycle arrest
In vitro/computational (Karan et al., 2020)	Cervical cancer	24h: 5–33 (μM)	Various cellular assays
In vitro/computational (Mayer et al., 2021)	Cervical cancer	–	Protein kinase inhibitor activity
In vitro/vivo (Lin et al., 2022)	Glioma	–	Cell cycle arrest/Apoptosis induction
In vitro (Wang et al., 2023)	Breast cancer	24h: 2.8–7.8 (μM)	Inhibition of EMT and autophagy via RIP1
In vitro/vivo (Lin et al., 2023)	Uterine leiomyoma	48h: 4.5 (μM)	Cell anti-proliferation
computational (Islam et al., 2023)	Colorectal cancer	–	–
computational (Horaira et al., 2023)	Colorectal cancer	–	–
In vitro/vivo (Karan et al., 2024)	Prostate cancer	48h: 3–6 (μM)	Blocking AR via E2F8

Missing IC_50_ values means these studies used MA concentrations based on published literature depending on the use of cell lines.

**Table 2:** Inhibitory activities of MA at 1 μM concentration against protein kinases.

Protein Kinase	% inhibition
MAPK-activated protein kinase-1a (MAPKAP-K1a/RSK-1)	68
Serum and glucocorticoid-induced kinase (SGK)	39
Lymphocyte kinase (LCK)	29
Mitogen and stress-activated protein kinase-1 (MSK1)	27
Glycogen synthase kinase-3β (GSK3β)	27
NIMA-related protein kinase 6 (NEK-6)	26
Stress-activated protein kinase-2a (SAPK2a/P38)	25
Protein kinase B (PKBα)	25
c-Jun N-terminal kinase (JNK-1)	20
p70 ribosomal protein S6 kinase (S6K1)	18
Dual tyrosine phosphorylated and regulated kinase 1A (DYRK1A)	18
Casein kinase-2 (CK2α)	17
Stress-activated protein kinase-2b (SAPK2b/p38b2)	16
Checkpoint kinase-1 (CHK1)	14
C-terminal Src Kinase (CSK)	11
MAPK kinase (mitogen-activated protein kinase) (MKK1)	10
cyclic AMP-dependent protein kinase (PKA)	10
protein kinase C (PKCα)	10

## Data Availability

All the inforamtion/data is included in the maniuscript
